# Job Satisfaction, Quality of Life, and Turnover Intention Among Nurses: A Comparative Study of Pattern-Based and Rotating Shift Schedules

**DOI:** 10.3390/healthcare13202551

**Published:** 2025-10-10

**Authors:** Yu Jin Jung, Haejin Kim

**Affiliations:** 1 Department of QPS (Quality and Patient Safety), Samsung Changwon Hospital, School of Medicine, Sungkyunkwan University, Changwon 51353, Republic of Korea; dbwlsdk2387@naver.com; 2 Department of Nursing, Changwon National University, 20 Changwondaehak-ro, Uichang-gu, Changwon 51140, Republic of Korea

**Keywords:** pattern-based work nurses, job satisfaction, quality of life, turnover intention

## Abstract

**Background/Objectives:** Shift work among nurses is associated with adverse outcomes, such as low job satisfaction, reduced quality of life, and high turnover intention. A pattern-based shift system has recently been introduced to provide more predictable and regular schedules. However, empirical research directly comparing the pattern-based shift system with traditional rotating shifts is lacking. Therefore, this study compared job satisfaction, quality of life, and turnover intention between nurses working under a pattern-based shift system and those working under a traditional rotating shift system. **Methods:** In total, 112 nurses (56 on a rotating shift and 56 on a pattern-based shift) were surveyed in this cross-sectional study. Job satisfaction was assessed using the Job Satisfaction Scale for Clinical Nurses, specifically developed for Korean nurses. Quality of life was measured using the Scale for Korean Adults’ Quality of Life. The Turnover Intention Measurement Tool, developed for Korean nurses, was used to evaluate turnover intention. **Results:** Nurses working under a pattern-based shift system reported significantly higher job satisfaction and quality of life than those in a traditional rotating shift system. No significant difference in turnover intention was observed between the two groups. **Conclusions:** Pattern-based shift systems were associated with higher job satisfaction and quality of life than traditional rotating shift systems; however, their impact on turnover intention was not significant. These findings highlight the need for comprehensive strategies in scheduling reform, as schedule predictability may improve nurses’ job satisfaction and quality of life but appears insufficient to reduce turnover intention, which is likely shaped by broader organizational and psychosocial factors.

## 1. Introduction

Nurses play an essential role as core personnel in medical services and take responsibility for patients’ health and safety. However, due to the 24 h operation of hospitals, they cannot avoid shift work, which can negatively impact their physical and mental health [1,2]. Irregular shift work and night work particularly disrupt biological rhythms and cause issues such as sleep deprivation, accumulated fatigue, and increased stress, which consequently lower job satisfaction [3,4,5]. Previous studies have shown that nurses who work in shifts experience relatively high levels of fatigue and emotional exhaustion, reducing work efficiency and affecting the quality of medical services [6,7]. This work pattern hinders the work–life balance of nurses and makes it challenging to reconcile their personal and professional lives [8,9,10]. Consequently, nurses’ job satisfaction decreases, and they tend to consider changing jobs in the long term [11,12,13]. Frequent changes in rotating shifts make it particularly difficult for nurses to maintain work–life balance. Several studies have reported that approximately 90–91% of night-shift nurses experience poor sleep quality, which in turn deteriorates their health [14,15]. Therefore, research on alternative work systems is needed, given the negative impact of shift work on nurses’ health and job performance.

In Korea, the Ministry of Health and Welfare has been implementing a pilot project since 2022 to improve nurses’ shift work systems as a policy initiative [16]. This initiative allows nurses to freely choose among various shift types—such as night-duty only, fixed single-shift, fixed two-shift, holiday-duty only, two-shift, and flexible working hours—to enable them to work under schedules that are more predictable and regular [17]. The pattern-based shift system was introduced under the government’s “Shift Work Improvement Project” as an alternative to the traditional rotating shift system [18]. Structurally, this system organizes rotations around night work in a fixed and recurring cycle. Nurses rotate across different groups—such as rotating, day-only, and night-only shifts—with equal opportunities for each type of assignment. This predictable and regular scheduling differentiates the pattern-based shift system from the traditional rotating shift system, in which assignments are irregular and subject to frequent changes [19].

Studies evaluating the government-led shift system in Korea’s improvement pilot project have reported that participation in the initiative enhanced nurses’ work–life balance and reduced turnover intention [17,20]. However, these studies analyzed participating wards as a single group without distinguishing between the different scheduling models included in the project. Therefore, empirical evidence regarding the unique effects of the pattern-based shift system remains limited. International research further underscores the importance of schedule regularity. Peršolja [21] demonstrated that cyclic schedules were associated with lower fatigue and improved recovery compared to non-cyclic schedules, while Al-Hammouri et al. [22] found that nurses working under fixed shifts reported significantly better sleep quality than those working under rotating shifts. These findings indicate that predictable scheduling provides benefits for nurses’ health and well-being; however, further studies are needed to directly compare the pattern-based shift system with the traditional rotating shift system in Korea.

The differences between the traditional rotating shift system and the pattern-based shift system can be interpreted through the Job Demands–Resources (JD-R) model [23]. This model explains how excessive job demands—such as irregular and unpredictable schedules, frequent night shifts, and heavy workload—drain workers’ resources, whereas job resources—including predictable schedules and recovery opportunities—can buffer negative effects and promote positive outcomes. Recent studies have emphasized that schedule predictability plays an important role in nursing. Chiang et al. [24] reported that fixed scheduling was associated with better sleep and lower stress levels, Emmanuel et al. [25] identified predictability as a key factor in supporting work–life balance, and Lim [26] highlighted the potential of structured pattern-based schedules in Korea. These findings suggest that the JD-R framework offers a useful lens for interpreting the potential influence of pattern-based scheduling on nurses’ work experiences.

Despite ongoing policy efforts, such as the Ministry of Health and Welfare’s “Shift Work Improvement Project,” systematic evaluations of alternative work systems in Korea remain limited. Pattern-based work systems are still in the pilot phase within nursing settings, and for new, improved systems to be widely adopted and stably implemented, their actual impact on nurses needs to be thoroughly assessed. Existing studies have suggested potential benefits, but did not distinguish between different scheduling models [17,20]. In particular, empirical research directly comparing the pattern-based shift system with the traditional rotating shift system is lacking. A comprehensive analysis of job satisfaction, quality of life, and turnover intention within the same framework is needed to capture the broader implications of scheduling reforms.

Therefore, this study aimed to provide basic data for improving shift work for nurses by comparing job satisfaction, quality of life, and turnover intention between nurses working under the existing rotational work system and those working under the pattern-based shift work system.

## 2. Materials and Methods

### 2.1. Design and Participants

This descriptive, cross-sectional study recruited nurses working under a rotating or pattern-based shift in a general hospital in Korea. In this study, the rotating shift system was defined as the traditional irregular three-shift schedule in which nurses individually rotate across morning, evening, and night shifts without fixed patterns, making it difficult to predict days off and work periods. The pattern-based shift system was defined as a scheduling method designed to enhance predictability by centering rotations around night shifts. Under this system, nurses rotate across different shift groups (rotating, day-only, and night-only shifts) in a regular cycle, with equal opportunities for each type of assignment.

The inclusion criteria were registered nurses employed in the hospital for at least 6 months, and those currently working under either a rotating shift or pattern-based shift system. Moreover, the exclusion criteria included nurses in administrative or managerial positions not directly engaged in clinical shift work, and those who submitted incomplete or invalid questionnaires.

The sample size was calculated using G*Power 3.1.9. program. Based on an independent t-test with a significance level of 0.05, an effect size of 0.50 (medium effect), and a power (1 − β) of 0.90, the minimum required sample size was determined to be 102 (51 per group). Considering a potential dropout rate of approximately 20%, a total of 122 questionnaires were distributed, and 122 were returned. A final sample of 112 responses (56 per group) was included in the analysis after excluding 10 incomplete responses.

### 2.2. Instruments

#### 2.2.1. General Characteristics

General characteristics included age, sex, marital status (single or married), religion (yes or no), education level (diploma, bachelor’s degree, or graduate degree), working unit (general ward or special unit such as operating room or intensive care unit [ICU]), years of work experience, and salary level. Participants selected the option that best represented their situation for each item.

#### 2.2.2. Job Satisfaction

Job satisfaction was measured using the Job Satisfaction Scale for Clinical Nurses, specifically developed and validated for Korean nurses by Lee et al. [27]. This tool comprises 33 items as follows: nine on organizational recognition and professional achievement, six on human maturity through the nursing profession, eight on interpersonal relationships of respect and recognition, four on fulfilling responsibilities as a nurse, three on demonstrating professional competence, and three on job stability and rewards. It uses a five-point Likert scale ranging from 1 (not at all) to 5 (very much), with a higher score indicating higher job satisfaction. The reliability of this tool was reported as Cronbach’s α = 0.95 in the development study [27]. In the present study, the reliability was Cronbach’s α = 0.96, indicating excellent internal consistency surpassing the conventional threshold of 0.70.

#### 2.2.3. Quality of Life

Quality of life was measured using the tool revised by Park [28], which is a supplemented version of the Scale for Korean Adults’ Quality of Life originally developed and validated in Korean populations by Yu [29] and Ro [30]. It comprises a total of 28 items, including seven on work life, five on self-esteem, six on emotional state, five on leisure activities, three on family relationships, and two on physical condition. Specifically, it is scored on a five-point Likert scale ranging from 1 to 5 points, with a higher score indicating a higher quality of life. Its reliability was reported as Cronbach’s α = 0.89 in the validation study by Park [28], while Cronbach’s α = 0.90 in this study, demonstrating strong internal consistency.

#### 2.2.4. Turnover Intentions

Turnover intention was measured using the Turnover Intention Measurement Tool developed by Yeun et al. [31], specifically designed for Korean clinical nurses. This tool comprises 10 items in the following three sub-areas: four on job satisfaction factors, three on work performance, and three on interpersonal relationships. The score was rated on a five-point Likert scale ranging from 1 (not at all) to 5 (very much), with a higher score indicating a higher turnover intention. This tool’s reliability was Cronbach’s α = 0.83 in the original development study [31], while Cronbach’s α = 0.86 in this study, confirming acceptable reliability levels.

### 2.3. Data Collection

Data were obtained from 17 to 28 April 2023. With the cooperation of the Nursing Department, the researcher visited units operating under both rotating and pattern-based shift systems and distributed questionnaires to the approved number of nurses in each unit, ensuring voluntary participation. Eligible nurses were those who understood the study purpose and provided written informed consent. After receiving the information sheet and consent form, participants completed the questionnaires, sealed them in envelopes with adhesive tape, and placed them in designated collection boxes within each unit. Subsequently, the researcher visited the ward and collected the questionnaires.

### 2.4. Data Analysis

Data were analyzed using IBM SPSS Statistics for Windows, version 28.0 (IBM Corp., Armonk, NY, USA). The participants’ general characteristics, job satisfaction, quality of life, and turnover intention were analyzed using frequencies, percentages, means, and standard deviations. Differences in general characteristics between the two groups were analyzed using the χ^2^ test, Fisher’s exact test, and an independent t-test. Independent t-tests and the Mann–Whitney U test were used to analyze the differences in job satisfaction, quality of life, and turnover intention between the two groups. Normality of continuous variables was assessed using the Kolmogorov–Smirnov test. Differences in job satisfaction, quality of life, and turnover intention according to the participants’ general characteristics were analyzed using independent t-tests and one-way analysis of variance. Post hoc tests were performed using the Scheffé test. All statistical analyses were performed with a significance level of 5%.

### 2.5. Ethical Consideration

This study was conducted in accordance with the principles of the Declaration of Helsinki. Before data collection, approval was obtained from the Institutional Review Board (IRB) of Samsung Changwon Hospital (IRB No.: 2022-12-014-003). All participants were informed about the study purpose and procedures, assured that their participation was voluntary, and informed that they could withdraw at any time without any disadvantage. Written informed consent was obtained from all participants before the distribution of the questionnaire. To ensure anonymity and confidentiality, completed questionnaires were sealed in envelopes by the participants and collected in designated boxes, which were later retrieved directly by the researcher. Data were used solely for research purposes.

## 3. Results

### 3.1. Differences in General Characteristics

No significant group differences were found across participants regarding their general characteristics (Table 1).

### 3.2. Differences in Job Satisfaction, Quality of Life, and Turnover Intention

Table 2 presents the differences in job satisfaction, quality of life, and turnover intention of nurses working under the rotating shift and pattern-based shift systems. Significant differences were found in job satisfaction and quality of life between the two groups, but not in turnover intention.

### 3.3. Differences in Job Satisfaction by Participants’ General Characteristics

In shift work, job satisfaction was significantly higher in married nurses than in single nurses (t = −2.62, *p* = 0.011). A significant difference was found in education level (F = 5.20, *p* = 0.009), and the results of the post-test showed that it was higher in nurses with a “graduate degree” than in those with a “bachelor’s degree.” Job satisfaction was significantly higher among nurses in the “general ward” than among those in the “special unit” (t = 2.53, *p* = 0.013), and a significant difference was found in salary levels (F = 8.49, *p* = 0.001; Table 3).

Conversely, job satisfaction of pattern-based shift nurses significantly differed only by length of service (F = 4.76, *p* = 0.013). Job satisfaction was higher in nurses with “≥5 years” of service than in those with “<2 years” of service (Table 3).

### 3.4. Differences in Quality of Life by Participants’ General Characteristics

In shift work, quality of life significantly differed according to educational level (F = 4.59, *p* = 0.015). The post hoc test results showed that it was higher among nurses with a “graduate degree” than among those with a “bachelor’s degree.” Quality of life also significantly differed by salary level (F = 9.43, *p* < 0.001). The post hoc test results showed that it was higher in nurses with a salary level of “≥3.5 million won” than in those with a salary level of “<2.5 million won” (Table 4).

In the pattern-based work type, quality of life was significantly higher in “married” nurses than in “single” nurses (t = −2.16, *p* = 0.036). Quality of life significantly differed by salary level (F = 3.79, *p* = 0.029). The post hoc test results showed that it was higher in nurses with a salary level of “≥3.5 million won” than in those with a salary level of “<2.5 million won”.

### 3.5. Differences in Turnover Intention by Participants’ General Characteristics

No significant differences were found in turnover intention according to general characteristics in either group (Table 5).

## 4. Discussion

This study compared job satisfaction, quality of life, and turnover intention between nurses working under a traditional rotating shift system and those in a pattern-based shift system. A key strength of this work is that it represents one of the few empirical studies directly examining pattern-based scheduling among clinical nurses. By focusing on outcomes central to both professional practice and workforce stability—job satisfaction, quality of life, and turnover intention—this study provides preliminary evidence on how alternative scheduling structures may be associated with nurses’ well-being. These findings contribute to ongoing discussions on work schedule reform in nursing practice and offer practical insights that may inform staffing strategies and policy decisions in clinical settings.

The results of this study indicated that pattern-based shift nurses reported significantly higher job satisfaction than rotating-shift nurses. Although direct comparisons are challenging due to the limited research on pattern-based shift systems, these findings align with previous evidence showing that regular two-shift nurses reported higher job satisfaction than irregular three-shift nurses [32]. Consistent with this, interviews with nurses who participated in a shift work improvement project in Korea highlighted that greater predictability contributed to improved job satisfaction and professional pride [20]. Recent studies further support these findings. Peršolja [21] demonstrated that cyclic schedules lead to better job-related outcomes than non-cyclic ones, while Emmanuel et al. [25] identified predictability and consistency as key shift attributes valued by nurses in maintaining work–life balance. In Korea, Lim [26] also underscored that predictable pattern-based schedules hold promise for improving nurses’ work experiences. Overall, these findings indicate that schedule predictability is a key determinant of nurses’ job satisfaction, warranting further empirical research to elucidate its underlying mechanisms.

Nurses working under the pattern-based shift system reported a significantly higher quality of life compared to those in rotating shifts, suggesting that schedule regularity supports daily routines, leisure activities, and social relationships. Consistent with this, Bang et al. [33] found that nurses who worked regular night shifts perceived their personal lives more positively than those in three-shift systems. Additional studies have also shown that rotating-shift nurses report lower life satisfaction, impaired family and social relationships, and fewer leisure opportunities compared with those on fixed daytime schedules [34,35]. Taken together, these findings indicate that schedule regularity may play an important role in shaping nurses’ perceived quality of life, and further research in diverse healthcare settings is needed to confirm whether the benefits observed in this study can be generalized more broadly.

These results can be interpreted within the framework of the JD-R model [23]. In this perspective, irregular rotating shifts impose high job demands—such as unpredictable schedules, night duties, and heavy workloads—that deplete nurses’ physical and psychological resources, lowering job satisfaction and quality of life. In contrast, the pattern-based shift system may serve as a job resource, offering greater predictability and recovery opportunities that buffer these negative effects. Recent studies support this view: Gou et al. [36] found that adequate resources mitigated the adverse impact of demanding schedules on ICU night-shift nurses, and Portoghese et al. [37] showed that nurses in low-demand/high-resource groups reported better well-being than those in high-demand/low-resource groups. Together, these findings suggest that predictable scheduling can function as a resource that supports nurses’ well-being even within demanding shift work environments.

In the present study, turnover intention did not significantly differ between the two groups of nurses. This finding contrasts with prior studies that reported significantly lower turnover intention among nurses participating in shift improvement projects compared to controls [17,20]. Such differences may reflect contextual factors, including the timing of implementation and hospital type. For instance, Kim et al. [20] surveyed nurses after two years of pilot project exposure, whereas the present study involved a shorter implementation period. Moreover, while our participants were primarily from general hospitals, Choi et al. [17] studied nurses mostly from tertiary hospitals (82.2%), where turnover rates are generally lower [38].

At the same time, previous research indicates that turnover intention is influenced not only by scheduling but also by indirect pathways involving fatigue, sleep quality, stress, and work–family conflict. For example, sleep disturbance has been shown to predict both turnover intention and actual turnover among shift-working nurses [39], and job stress and sleep disturbance significantly increased turnover intention among new graduates [40]. Work–family conflict also exerts direct and indirect effects through psychological distress or emotional states [41], while burnout mediates the impact of multiple job demands on turnover intention within the JD-R framework [42].

Taken together, the null finding in this study may reflect the persistence of these unmitigated demands despite the addition of predictable scheduling. Given the limitations of the bivariate analyses, future research should adopt longitudinal and multivariate designs that incorporate mediating and moderating variables—such as fatigue, stress, sleep quality, and work–family conflict—to clarify the mechanisms linking scheduling reforms and turnover outcomes.

When job satisfaction was compared by general characteristics, significant differences were observed among rotating-shift nurses according to marital status, education level, working unit, and salary level. However, differences only emerged according to work experience among pattern-based shift nurses. This suggests that job satisfaction among rotating-shift nurses is more sensitive to several personal and environmental factors, whereas the predictability of the pattern-based schedule can mitigate some of these influences. Previous research has also indicated that irregular scheduling amplifies the influence of contextual factors such as marital and educational statuses on satisfaction levels [43,44,45]. Therefore, pattern-based scheduling could be adopted as a strategy for reducing disparities in satisfaction and promoting a more stable and supportive work environment.

Quality of life also showed notable patterns when compared by participants’ general characteristics. In both groups, salary level was significantly related to quality of life, a finding consistent with previous studies, which reported that financial compensation plays a central role in nurses’ well-being [46,47]. Within the pattern-based group, single nurses reported a higher quality of life than married nurses, possibly reflecting reduced domestic responsibilities and greater autonomy in the use of non-working time [17,48]. These findings indicate that while schedule predictability generally benefits nurses’ quality of life, its impact can be perceived differently depending on individual life circumstances, such as marital status or caregiving responsibilities. Therefore, work scheduling reforms may be more effective if designed with flexibility and sensitivity to personal contexts, allowing diverse groups of nurses to experience equitable improvements in their well-being.

Finally, no general characteristics showed significant differences in turnover intentions between the groups. These results align with those of previous studies reporting that turnover intention is more influenced by structural factors—such as work environment, organizational culture, job stress, and the burden of nursing work—than individual sociodemographic characteristics [49,50]. It can be inferred that turnover intention is closely associated with overall working conditions, such as long working hours, fatigue, insufficient human resource support, and a low level of organizational support, rather than simply individual characteristics. Therefore, reducing nurses’ turnover intention requires improvements in individual factors and the simultaneous implementation of organizational-level approaches.

### Limitations and Future Research

This study had some limitations that should be noted. The study was conducted in a single hospital, which may restrict the findings’ generalizability to other healthcare institutions. The pattern-based work system was also in a pilot stage during the study period, and nurses’ expectations or uncertainties about the system may have influenced their responses. Importantly, this study did not control for all potential confounders, such as hospital size, unit workload, nurse-to-patient ratio, leadership style, or organizational culture. The absence of these variables may have influenced the outcomes and limited the validity of direct comparisons between shift types. Furthermore, the statistical analyses relied on t-tests and one-way analysis of variance rather than multivariate models, which limited the ability to control covariates and fully account for potential interactions. The reliance on Likert-type ordinal scales analyzed with parametric tests assumes equal intervals, which may not strictly hold true. These limitations highlight the need for careful interpretation of the findings.

Future research is needed to build on these findings and address current limitations. Multi-site studies with larger and more diverse nurse populations, using longitudinal designs, would enhance generalizability and clarify the long-term impact of pattern-based work systems, including adaptation processes and organizational stability. Also, future research should test mediation and moderation models to clarify whether schedule predictability buffers the negative effects of fatigue, stress, or work–life conflict on turnover outcomes. Incorporating multivariate approaches such as regression or analysis of covariance will allow us control over covariates and facilitate more robust inferences regarding the effects of scheduling systems. Moreover, expanding outcomes beyond individual well-being to include patient safety, quality of care, and nurse retention will provide a broader evaluation of system effectiveness. Finally, studies guided by the JD–R model that integrate job demands (e.g., workload intensity and time pressure), job resources (e.g., schedule predictability and supervisor support), and personal resources (e.g., resilience and self-efficacy) can provide a more comprehensive understanding of the mechanisms through which pattern-based scheduling shapes nurses’ work experiences and organizational sustainability.

## 5. Conclusions

This study found that nurses working under a pattern-based shift system reported significantly higher job satisfaction and quality of life than those working under a traditional rotating shift system. No significant difference was observed in turnover intention. When job satisfaction was compared according to participants’ general characteristics, rotating-shift nurses showed significant differences in terms of marital status, education level, working unit, and salary level. Differences were observed only by work experience among nurses in the pattern-based group. Quality of life was significantly associated with salary level in both groups. Within the pattern-based shift group, single nurses reported a higher quality of life than married nurses. No significant differences in turnover intention were found according to general characteristics in either group. These findings provide preliminary evidence that pattern-based scheduling may support nurses’ well-being; however, further longitudinal studies are needed to clarify its long-term impact and organizational implications.

## Figures and Tables

**Table 1:** Differences in general characteristics between the two groups.

Characteristics	Categories	Shift Work (*n* = 56)	Pattern Work (*n* = 56)	χ^2^/t	*p*
		*n* (%)	*n* (%)		
Age (years)	≤24	9 (16.1)	10 (17.9)	1.12	0.572
	25–29	29 (51.8)	33 (58.9)		
	≥30	18 (32.1)	13 (23.2)		
	M ± SD	29.66 ± 6.25	28.77 ± 6.56	0.74	0.462
Sex	Male	1 (1.8)	2 (3.6)	-	1.00 ^†^
	Female	55 (98.2)	54 (96.4)		
Marital status	Single	45 (80.4)	42 (75.0)	0.21	0.650
	Married	11 (19.6)	14 (25.0)		
Religion	Yes	11 (19.6)	12 (21.4)	0.00	1.00
	No	45 (80.4)	44 (78.6)		
Education	Diploma	5 (10.7)	3 (5.4)	-	0.617 ^†^
	Bachelor’s degree	46 (82.1)	50 (89.3)		
	Graduate degree	4 (7.2)	3 (5.4)		
Working unit	General ward	21 (37.5)	27 (48.2)	0.91	0.340
	Special unit	35 (62.5)	29 (51.8)		
Work experience (years)	<2	14 (25.0)	18 (32.2)	1.43	0.539
	2 < 5	17 (30.4)	19 (33.9)		
	≥5	25 (44.6)	19 (33.9)		
	M ± SD	6.09 ± 6.09	5.70 ± 6.77	0.33	0.745
Salary level	<2.5 million won	10 (17.9)	8 (14.3)	0.62	0.769
	2.5–3.5 million won	42 (75.0)	42 (75.0)		
	≥3.5 million won	4 (7.1)	6 (10.7)		

Note: M, mean; SD, standard deviation; ^†^, Fisher’s exact test.

**Table 2:** Differences in job satisfaction, quality of life, and turnover intention between the two groups.

Variable	Shift Work (*n* = 56)	Pattern Work (*n* = 56)	t/z	*p*
	M ± SD	M ± SD		
Job satisfaction	3.33 ± 0.54	3.63 ± 0.39	−3.85	<0.001 ^†^
Quality of life	2.86 ± 0.46	3.20 ± 0.42	−3.88	<0.001
Turnover intention	3.94 ± 0.57	3.75 ± 0.64	−1.52	0.129 ^†^

Note: M, mean; SD, standard deviation; ^†^, Mann–Whitney U test.

**Table 3:** Differences in job satisfaction by participants’ general characteristics.

Characteristics	Categories	Shift Work (*n* = 56)			Pattern Work (*n* = 56)		
		M ± SD	F/t	*p* (Scheffe)	M ± SD	F/t	*p* (Scheffe)
Age (years)	≤24	3.21 ± 0.45	2.17	0.124	3.47 ± 0.40	2.48	0.094
	25–29	3.23 ± 0.49			3.61 ± 0.40		
	≥30	3.54 ± 0.62			3.83 ± 0.32		
Sex	Male	2.48 ± 0.00	−1.58	0.119	3.67 ± 0.81	0.12	0.903
	Female	3.34 ± 0.54			3.63 ± 0.38		
Marital status	Single	3.24 ± 0.49	−2.62	0.011	3.57 ± 0.40	−2.00	0.050
	Married	3.69 ± 0.62			3.81 ± 0.32		
Religion	Yes	3.40 ± 0.52	0.52	0.607	3.66 ± 0.38	−0.26	0.798
	No	3.31 ± 0.55			3.63 ± 0.40		
Education	Diploma ^a^	3.56 ± 0.64	5.20	0.009 (^b^ < ^c^)	3.75 ± 0.05	0.37	0.696
	Bachelor’s degree ^b^	3.23 ± 0.48			3.63 ± 0.39		
	Graduate degree ^c^	4.02 ± 0.58			3.78 ± 0.63		
Working unit	General ward	3.56 ± 0.54	2.58	0.013	3.71 ± 0.32	1.36	0.181
	Special unit	3.19 ± 0.50			3.57 ± 0.44		
Work experience (years)	<2 ^a^	3.35 ± 0.59	0.08	0.923	3.43 ± 0.48	4.76	0.013 (^a^ < ^c^)
	2 < 5 ^b^	3.28 ± 0.53			3.66 ± 0.31		
	≥5 ^c^	3.34 ± 0.55			3.80 ± 0.29		
Salary level	<2.5 million won ^a^	3.03 ± 0.55	8.49	0.001 (^a, b^ < ^c^)	3.54 ± 0.45	2.49	0.093
	2.5–3.5 million won ^b^	3.31 ± 0.45			3.60 ± 0.39		
	≥3.5 million won ^c^	4.20 ± 0.61			3.95 ± 0.19		

Note: M, mean; SD, standard deviation. Superscript letters (a–c) denote post-hoc groupings within each comparison; values that share a letter are not significantly different, whereas values with different letters differ significantly (Scheffé’s post hoc test, *p* < 0.05). Letters are assigned independently within each variable and do not represent the same group across different variables.

**Table 4:** Differences in quality of life by participants’ general characteristics.

Characteristics	Categories	Shift Work (*n* = 56)			Pattern Work (*n* = 56)		
		M ± SD	F/t	*p* (Scheffe)	M ± SD	F/t	*p* (Scheffe)
Age (years)	≤24	2.83 ± 0.28	2.38	0.101	3.20 ± 0.56	1.94	0.173
	25–29	2.75 ± 0.46			3.12 ± 0.34		
	≥30	3.05 ± 0.50			3.40 ± 0.47		
Sex	Male	3.21 ± 0.00	0.76	0.448	3.30 ± 0.68	0.35	0.726
	Female	2.86 ± 0.47			3.20 ± 0.42		
Marital status	Single	2.80 ± 0.40	−1.98	0.053	3.13 ± 0.40	−2.16	0.036
	Married	3.10 ± 0.62			3.40 ± 0.44		
Religion	Yes	2.71 ± 0.47	−1.18	0.244	3.09 ± 0.37	−1.00	0.324
	No	2.90 ± 0.46			3.23 ± 0.43		
Education	Diploma ^a^	3.08 ± 0.40	4.59	0.015 (^b^ < ^c^)	3.11 ± 0.29	0.79	0.459
	Bachelor’s degree ^b^	2.79 ± 0.42			3.19 ± 0.43		
	Graduate degree ^c^	3.41 ± 0.69			3.49 ± 0.46		
Working unit	General ward	2.97 ± 0.49	1.33	0.188	3.13 ± 0.43	−1.18	0.243
	Special unit	2.80 ± 0.44			3.26 ± 0.41		
Work experience (years)	<2 ^a^	2.87 ± 0.44	0.15	0.864	3.22 ± 0.48	2.10	0.133
	2 < 5 ^b^	2.81 ± 0.46			3.05 ± 0.34		
	≥5 ^c^	2.89 ± 0.50			3.33 ± 0.42		
Salary level	<2.5 million won ^a^	2.66 ± 0.36	9.43	<0.001 (^a, b^ < ^c^)	2.96 ± 0.47	3.79	0.029 (^a^ < ^c^)
	2.5–3.5 million won ^b^	2.83 ± 0.42			3.19 ± 0.38		
	≥3.5 million won ^c^	3.68 ± 0.38			3.56 ± 0.48		

Note: M, mean; SD, standard deviation. Superscript letters (a–c) denote post-hoc groupings within each comparison; values that share a letter are not significantly different, whereas values with different letters differ significantly (Scheffé’s post hoc test, *p* < 0.05). Letters are assigned independently within each variable and do not represent the same group across different variables.

**Table 5:** Differences in turnover intention by participants’ general characteristics.

Characteristics	Categories	Shift Work (*n* = 56)			Pattern Work (*n* = 56)		
		M ± SD	F/t	*p* (Scheffe)	M ± SD	F/t	*p* (Scheffe)
Age (years)	≤24	3.84 ± 0.29	0.17	0.841	3.50 ± 0.93	3.12	0.068
	25–29	3.94 ± 0.68			3.92 ± 0.53		
	≥30	3.98 ± 0.49			3.51 ± 0.54		
Sex	Male	3.50 ± 0.00	−0.77	0.442	3.70 ± 0.57	−0.11	0.915
	Female	3.95 ± 0.57			3.75 ± 0.65		
Marital status	Single	3.91 ± 0.62	−0.70	0.487	3.82 ± 0.65	1.55	0.127
	Married	4.05 ± 0.31			3.53 ± 0.56		
Religion	Yes	3.95 ± 0.38	0.11	0.913	3.75 ± 0.62	0.01	0.991
	No	3.93 ± 0.61			3.75 ± 0.65		
Education	Diploma	3.98 ± 0.53	0.27	0.768	3.93 ± 0.67	1.27	0.290
	Bachelor’s degree	3.92 ± 0.60			3.77 ± 0.63		
	Graduate degree	4.13 ± 0.30			3.20 ± 0.72		
Working unit	General ward	4.08 ± 0.31	1.69	0.159	3.88 ± 0.64	1.48	0.145
	Special unit	3.85 ± 0.67			3.63 ± 0.62		
Work experience (years)	<2	3.85 ± 0.28	0.62	0.541	3.68 ± 0.68	0.42	0.658
	2 < 5	3.87 ± 0.73			3.86 ± 0.66		
	≥5	4.03 ± 0.57			3.71 ± 0.60		
Salary level	<2.5 million won	3.97 ± 0.51	0.06	0.940	3.85 ± 0.26	0.51	0.614
	2.5–3.5 million won	3.94 ± 0.53			3.75 ± 0.68		
	≥3.5 million won	3.85 ± 1.14			3.57 ± 0.74		

Note: M, mean; SD, standard deviation.

## Data Availability

The dataset used and analyzed in this study is available from the corresponding author. The data are not publicly available due to privacy concerns.

## Abbreviations

The following abbreviations are used in this manuscript:

ICU	Intensive care unit.
J-DR	Job Demands–Resources.
